# Quantification of Marine Picocyanobacteria on Water Column Particles and in Sediments Using Real-Time PCR Reveals Their Role in Carbon Export

**DOI:** 10.1128/msphere.00499-22

**Published:** 2022-12-06

**Authors:** Jiandong Zhang, Furun Li, Lijuan Long, Sijun Huang

**Affiliations:** a CAS Key Laboratory of Tropical Marine Bio-resources and Ecology, South China Sea Institute of Oceanology, Chinese Academy of Sciences, Guangzhou, China; b Marine Environmental Engineering Center, South China Sea Institute of Oceanology, Chinese Academy of Sciences, Guangzhou, China; c Southern Marine Science and Engineering Guangdong Laboratory (Guangzhou), Guangzhou, China; d University of Chinese Academy of Sciences, Beijing, China; University of Wisconsin-Madison

**Keywords:** picocyanobacteria, qPCR, quantification, carbon export, biological carbon pump, new primers, 16S-23S rRNA ITS

## Abstract

Picocyanobacteria are the most abundant primary producers in the ocean and play a fundamental role in marine carbon cycling. Quantification of picocyanobacteria on sinking particles and in sediments is essential to understanding their contribution to the biological carbon pump. We designed a primer set targeting the 16S-23S rRNA internal transcribed spacer (ITS) sequence of cyanobacteria and established a quantitative PCR (qPCR) method for quantifying the ITS sequence abundance. High-throughput sequencing confirmed that this primer set can cover broad diversities of marine picocyanobacteria and avoid amplification of other marine cyanobacteria such as *Trichodesmium* and *Crocosphaera*. Amplification efficiencies were slightly different when seven marine *Synechococcus* and *Prochlorococcus* strains were assayed. The qPCR results were comparable with flow cytometry for water samples. Using this method, we found that, in the dark ocean, picocyanobacterial ITS sequence abundances were 10 to 100 copies/mL in the size fraction of 0.2 to 3 μm, which were 1 to 3 orders of magnitude more abundant than on the >3-μm particles. We also found that picocyanobacterial ITS abundance in sediment ranged from 10^5^ to 10^7^ copies/g along two nearshore-to-offshore transects in the northern South China Sea. These results further explain the important role of picocyanobacteria in carbon export. Collectively, we provide a qPCR method quantifying the total abundance of marine picocyanobacteria on water column particles and in sediments. Moreover, this newly designed primer set can be also applied to investigate the community of picocyanobacteria via high-throughput sequencing.

**IMPORTANCE** Picocyanobacteria are the most abundant primary producers in the ocean. However, quantification of picocyanobacteria on the sinking particles and in sediments remains challenging using flow cytometry or epifluorescence microscopy. Here, we developed a real-time PCR method to quantify picocyanobacteria using a newly designed primer set specifically targeting the 16S-23S rRNA ITS sequence of cyanobacteria. We showed that in the dark ocean, picocyanobacteria are 1 to 3 orders of magnitude more abundant in small particles (0.2 to 3 μm) than in larger particles (>3 μm). This result supports the important role of direct sinking free-living picocyanobacteria cells in the carbon export to deep ocean. We also found that the picocyanobacterial ITS sequence abundance were 10^5^ to 10^7^ copies per gram in sediments, suggesting significant accumulation of sinking picocyanobacteria in the benthic ecosystem. This qPCR method can be used to quantify the contribution of picocyanobacteria to the biological carbon pump.

## INTRODUCTION

Picocyanobacteria are the most abundant phototrophs in the ocean, responsible for 50% of the primary production in the oligotrophic area (1), with *Synechococcus* and *Prochlorococcus* being the two major genera (2–4). Based on the 16S rRNA gene phylogeny, *Synechococcus* spp. are divided into 5 clusters, and marine *Synechococcus* are affiliated with cluster 5 (5). Cluster 5 *Synechococcus* also encompasses many nonmarine members, such as those from lakes (6). *Synechococcus* in cluster 5, *Prochlorococcus*, and *Cyanobium* together form a robust, coherent phylogenetic clade, designated the “Syn/Pro clade” (7) or “PS clade” (8). Because of their tiny cell size, marine picocyanobacteria were thought to be resistant to sink, which limits their contribution in the biological carbon pump (9, 10). However, picocyanobacteria were frequently found on sinking particles and in sediments (11–13), and they were considered to represent a significant contribution to carbon export to deep ocean (14). Therefore, quantification of picocyanobacteria on sedimenting particles and in sediment is essential to understand their role in carbon export.

To quantify marine picocyanobacteria *Synechococcus* and *Prochlorococcus*, several methods have been developed, including epifluorescence microscopy (EFM) (3), flow cytometry (FCM) (15), peptide nucleic acid (PNA) probe-based *in situ* rRNA hybridization (16), dot blot hybridization (17–20), real-time quantitative PCR (qPCR) (21), and measurement of chlorophylls and carotenoid pigments through high-performance liquid chromatography (HPLC) (22). These approaches can be divided into three groups, cell counting based on autofluorescence, molecular enumeration, and pigment measurement. Direct and indirect (based on autofluorescence and pigment) picocyanobacterial cell counting by EFM and FCM targets the entire community. Molecular approaches such as qPCR and dot blot hybridization can discriminate between different ecotypes of *Prochlorococcus* and *Synechococcus* and therefore can provide much more detailed quantitative information.

Although FCM is an accurate, quick method to quantify cell numbers of the community of picocyanobacteria, it has a few limitations. First, gating and then counting on an FCM plot is difficult when picocyanobacteria cell abundance is extremely low, especially for samples from the base or below the euphotic zone. Second, the picocyanobacteria can aggregate in the sinking particles, which are composed of both organisms (e.g., phytoplankton and zooplankton) and detrital material (e.g., fecal pellets and aggregates). For particles and sediments, FCM is not applicable to count picocyanobacteria cells. In contrast, EFM can be used to enumerate picocyanobacteria cells in sediment or associated with particles (12, 13, 23). However, counting by EFM involves complicated sediment sample treatment and suffers from low throughput.

The qPCR method has previously been used to quantify ecotypes of *Prochlorococcus* (21, 24, 25) and marine *Synechococcus* (26–28). Using this method, ocean-scale distribution patterns (24, 28) and temporal variations (26, 29) of different *Prochlorococcus* and *Synechococcus* ecotypes have been described. Moreover, qPCR was also applied to quantify other specific picocyanobacteria clades in freshwater lakes (30). A few cyanobacterium-specific primers targeting the 16S rRNA gene have been designed to detect cyanobacteria in nature, such as CYA106F and CYA781R (31), and OXY107F and OXY1313R (18). Two primer sets (CYAN108F-CYAN377R and CYA359F-CYA781R) have been used for qPCR to quantify the total cyanobacteria community in lakes (32, 33). However, thus far, qPCR has not been used to specifically quantify the entire community of marine picocyanobacteria, i.e., marine *Synechococcus* and *Prochlorococcus*.

To quantify the entire community of marine picocyanobacteria, we designed a primer set that targets the 16S-23S rRNA internal transcribed spacer (ITS) sequence of cyanobacteria. Based on the qPCR method, a standard curve was established to quantify the abundance of picocyanobacteria in water columns and surface sediments. The newly developed method was then assessed by comparing it to FCM using the same set of water samples. Using this method, picocyanobacterial abundance on particles with different size fractions and in sediment were assayed. Finally, details and a database were provided to analyze the diversity and community structure of marine picocyanobacteria via high-throughput amplicon sequencing using this primer set.

## RESULTS

### Design of primers, amplification efficiency, diversity coverage, and specificity.

The forward primer Picocya-Ala-F, 5′-GCTTTGCAAGCAGGATGTCAG-3′, which targets the tRNA Ala, has a perfect matching on members of marine picocyanobacteria. This forward primer also perfectly matches a few freshwater cyanobacteria. However, there are five mismatches in the sequence of marine nitrogen-fixing cyanobacteria *Trichodesmium*. We also found that a highly conserved antitermination box A motif exists in the ITS sequences of all known marine picocyanobacteria strains and designed the reverse primer as Picocya-boxA-R, 5′-CTATGCAGTTGTCAAGGTTC-3′. This conserved motif also exists in a few freshwater cyanobacteria but is absent in the nitrogen-fixing marine cyanobacterium *Crocosphaera*. Collectively, this primer set presumably covers a broad diversity of marine picocyanobacteria within the “Syn/Pro clade” and avoids amplifying the dominant marine nitrogen-fixing cyanobacteria.

To illustrate the diversity coverage and specificity, the primer set was tested using genomic DNA from *Synechococcus*, *Prochlorococcus*, *Trichodesmium*, *Crocosphaera*, and a few freshwater cyanobacteria (strains are shown in Table 1). Except for the non-Syn/Pro clade strain *Synechococcus* sp. PCC7002 and the marine nitrogen-fixing cyanobacteria Trichodesmium erythraeum IMS101 and Crocosphaera watsonii WH8501, all other tested strains resulted in successful PCR amplification.

**TABLE 1:** Cyanobacteria strains or synthesized ITS sequences used in this study^*a*^

Genus or species	Strain	Ecotype	Amplicon size (bp)	Source	Amplification efficiency	Used for standard curve
*Synechococcus*	CC9311	Sub5.1, clade I	256	gDNA	93.78 %	Yes
*Synechococcus*	YX02-1	Sub5.1, clade II	257	gDNA	90.87 %	Yes
*Synechococcus*	WH7803	Sub5.1, clade V	263	gDNA	94.1%	Yes
*Synechococcus*	WH7805	Sub5.1, clade VI	269	gDNA	99.95 %	Yes
*Synechococcus*	CB0101	Sub5.2, clade CB4	271	gDNA	102.6 %	Yes
*Prochlorococcus*	MED4	HLI	203	gDNA	92.08 %	Yes
*Prochlorococcus*	MIT9312	HLII	194	Synthesized sequence	NA	Yes
*Prochlorococcus*	NATL1A	LLI	215	gDNA	93.86 %	Yes
*Prochlorococcus*	MIT9313	LLIV	239	Synthesized sequence	NA	Yes
*Synechococcus*	PCC7002	Non-Syn/Pro clade		gDNA	NA	
Trichodesmium erythraeum	IMS101	Non-Syn/Pro clade		gDNA	NA	
Crocosphaera watsonii	WH8501	Non-Syn/Pro clade		gDNA	NA	
*Synechococcus*	FACHB-1061	Non-Syn/Pro clade	162	gDNA	NA	
*Synechococcus*	FACHB-410	Non-Syn/Pro clade	119	gDNA	NA	
Leptolyngbya boryana	FACHB-2210	Non-Syn/Pro clade	180	gDNA	NA	
Richelia sinica	FACHB-800	Non-Syn/Pro clade	124	gDNA	NA	
Planktothrix agardhii	FACHB-920	Non-Syn/Pro clade	141	gDNA	NA	

a NA, not analyzed; gDNA, genomic DNA.

**FIG 1:**
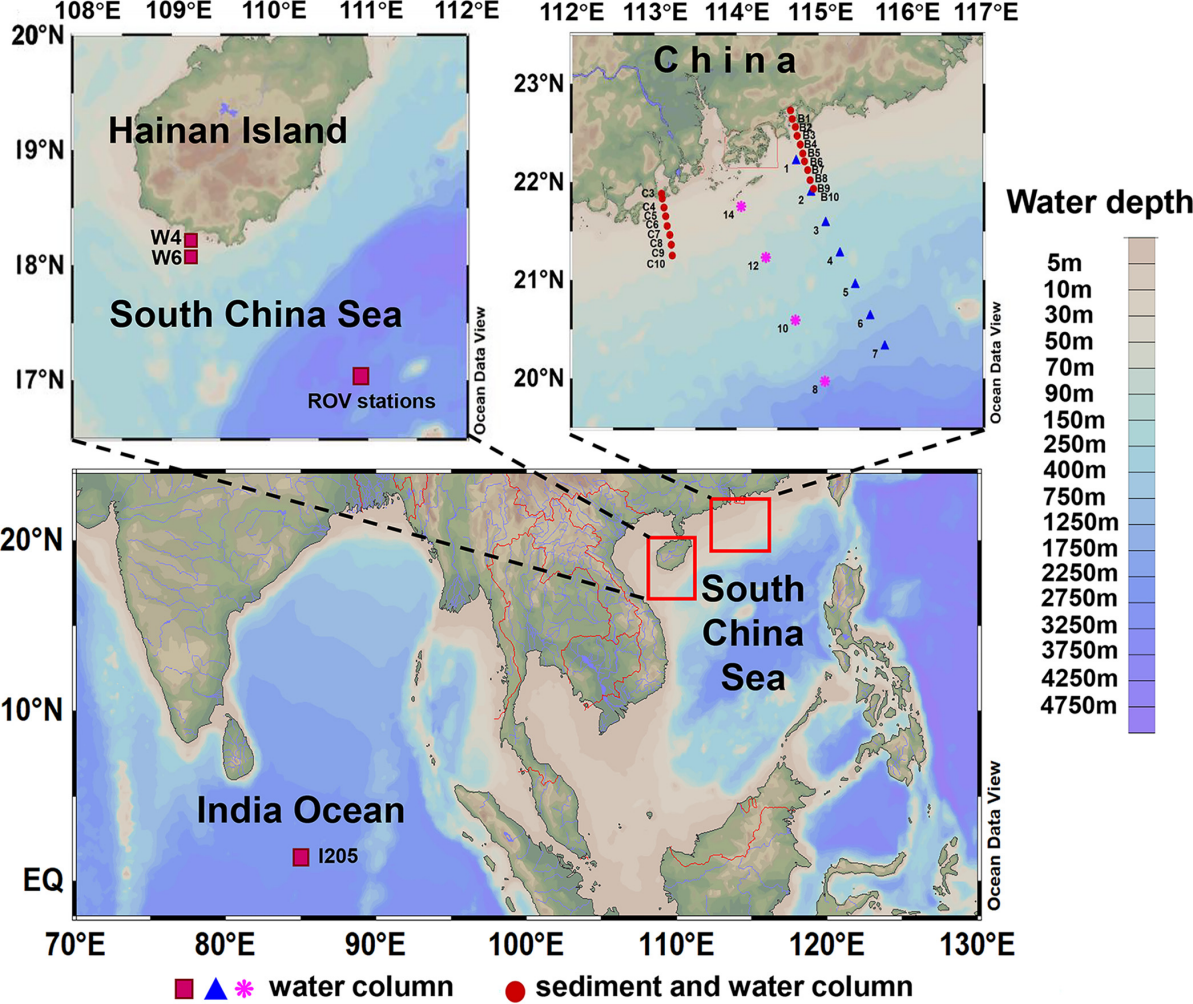
Sampling sites in the South China Sea. Blue triangle, water samples for FCM counting and qPCR assay; red circle, water and sediment samples for qPCR assay; red square, water samples of different size fractions for qPCR assay; pink asterisks, water samples for diversity of the picocyanobacteria. Three ROV stations were geographically very close to each other, with a distance of less than 2 km.

Environmental DNA from the coastal Sanya Bay (stations W4 and W6) (Fig. 1) and an Indian Ocean water column (Fig. 1, station I205) were also tested, and four clone libraries were built. Cloned sequences from the four libraries all fell into the Syn/Pro clade of cyanobacteria (see Fig. S1 in the supplemental material). Clade II *Synechococcus* within the subcluster 5.1 dominated the Sanya Bay community, and *Prochlorococcus* of high-light-adapted (HL) ecotype HLII prevailed in the upper layer (5 to 25 m) of the Indian Ocean station, while low-light-adapted (LL) ecotypes were prevalent in the middle to lower euphotic zone (50 to 150 m). LL *Prochlorococcus* sequences retrieved were highly divergent such that 25 out of 59 LL sequences did not cluster with LL ecotypes LLI to LLIV and thus were denoted as LL-NC. These results indicate that amplification for marine samples using this primer set specifically targets the Syn/Pro clade and covers the broad diversity within this group of marine picocyanobacteria.

10.1128/msphere.00499-22.1 FIG S1 Phylogenetic diversity of picocyanobacteria assessed by using the primers designed in this study. Samples were collected from the coastal water of Sanya Bay, South China Sea, and the oceanic water in the Indian Ocean (station I205). The phylogenetic tree was constructed by MEGA 7 using the maximum-likelihood method and visualized in iTOL. The bootstrap test was performed for 100 replicates. Download FIG S1, TIF file, 1.1 MB. Copyright © 2022 Zhang et al. 2022 Zhang et al. https://creativecommons.org/licenses/by/4.0/ This content is distributed under the terms of the Creative Commons Attribution 4.0 International license.

**FIG 2:**
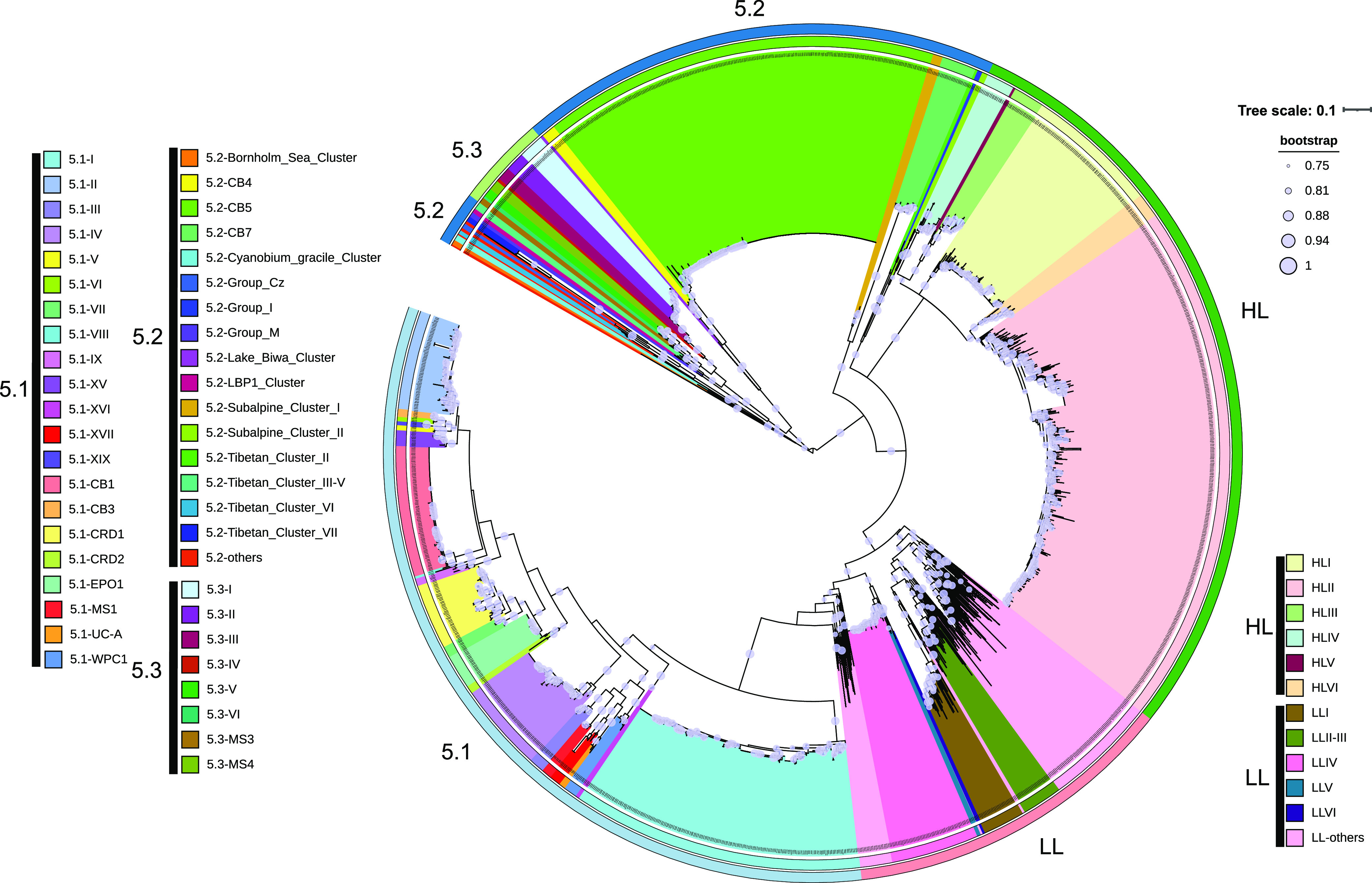
Phylogenetic tree showing the ecotypes of picocyanobacteria based on 1,699 ITS sequences which were used as reference sequences for high-throughput ITS amplicon sequencing data analysis. All of the known ecotypes within the Syn/Pro clade were involved. The *Synechococcus* ecotype 5.1-EPO1 was newly defined in this study, within which the environmental sequences were first found in the equatorial Pacific Ocean and assigned to ecotype CRD1. The tree was built using FastTree implemented in QIIME2. The bootstrap test was performed for 1,000 replicates.

**FIG 3:**
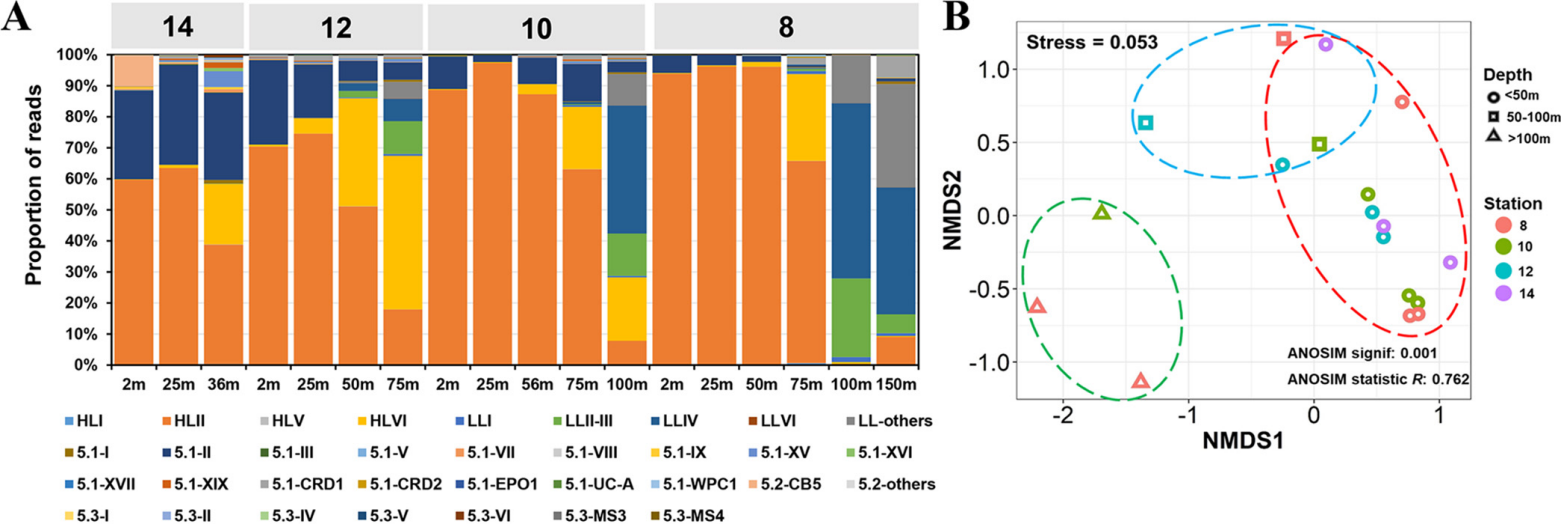
Community analysis of picocyanobacteria along a South China Sea transect via high-throughput amplicon sequencing. (A) Relative abundances of ecotypes within each sample. (B) Nonmetric dimensional scaling showing the community similarity among samples.

We also established the method for analyzing diversity and community composition of marine picocyanobacteria with high-throughput sequencing using this primer set. We first collected 1,699 ITS sequences within the Syn/Pro clade and identified their phylogenetic affiliation with picocyanobacteria ecotypes. We also collected 669 cyanobacterial ITS sequences not belonging to the Syn/Pro clade, using them to filter out reads not from the Syn/Pro clade. We built up a reference database containing those 1,699 plus 669 cyanobacterial ITS sequences for high-throughput sequence processing. This database covers all the known ecotypes within the Syn/Pro clade of picocyanobacteria (Fig. 2). Using the primer set and the database, we analyzed the samples from a South China Sea transect encompassing stations 8, 10, 12, and 14 (Fig. 1). A total of 1,181,301 clean reads were generated by using the MiSeq platform. After denoise using DADA2, 1,044,471 reads (88.4%) were retained, and then, after specificity filtration and annotation, 1,040,074 reads (88%) were affiliated with ecotypes. There were no reads that were assigned to the non-Syn/Pro clade. These data indicated that only very few reads (0.4%) were filtered out due to nonspecific amplification for these marine samples. Among these samples in the transect, *Prochlorococcus* ecotypes HLII and HLVI dominated in the upper euphotic zone (<75 m), and LL ecotypes dominated in the lower euphotic zone (100 to 150 m) (Fig. 3A). It is interesting that the HLVI ecotype was prevalent in the middle layer of the euphotic zone, confirming the previous finding that HLVI is likely an “intermediate ecotype” (34). The nonmetric dimensional scaling diagram shows a clear pattern that samples were grouped according to sampling depth (Fig. 3B). Consistent with the community analysis using the clone library, high-throughput amplicon sequencing also confirmed that this primer set is useful for analyzing marine picocyanobacteria. It is worth noting that *Synechococcus* strains in subcluster 5.2 harbor a vast unexplored diversity, and they are prevalent in estuarine and nearshore waters. The database should be expanded based on the local data when analyzing estuarine and nearshore samples.

### Amplification efficiency, standard curve, repeatability, reproducibility, and detection limitation.

**FIG 4:**
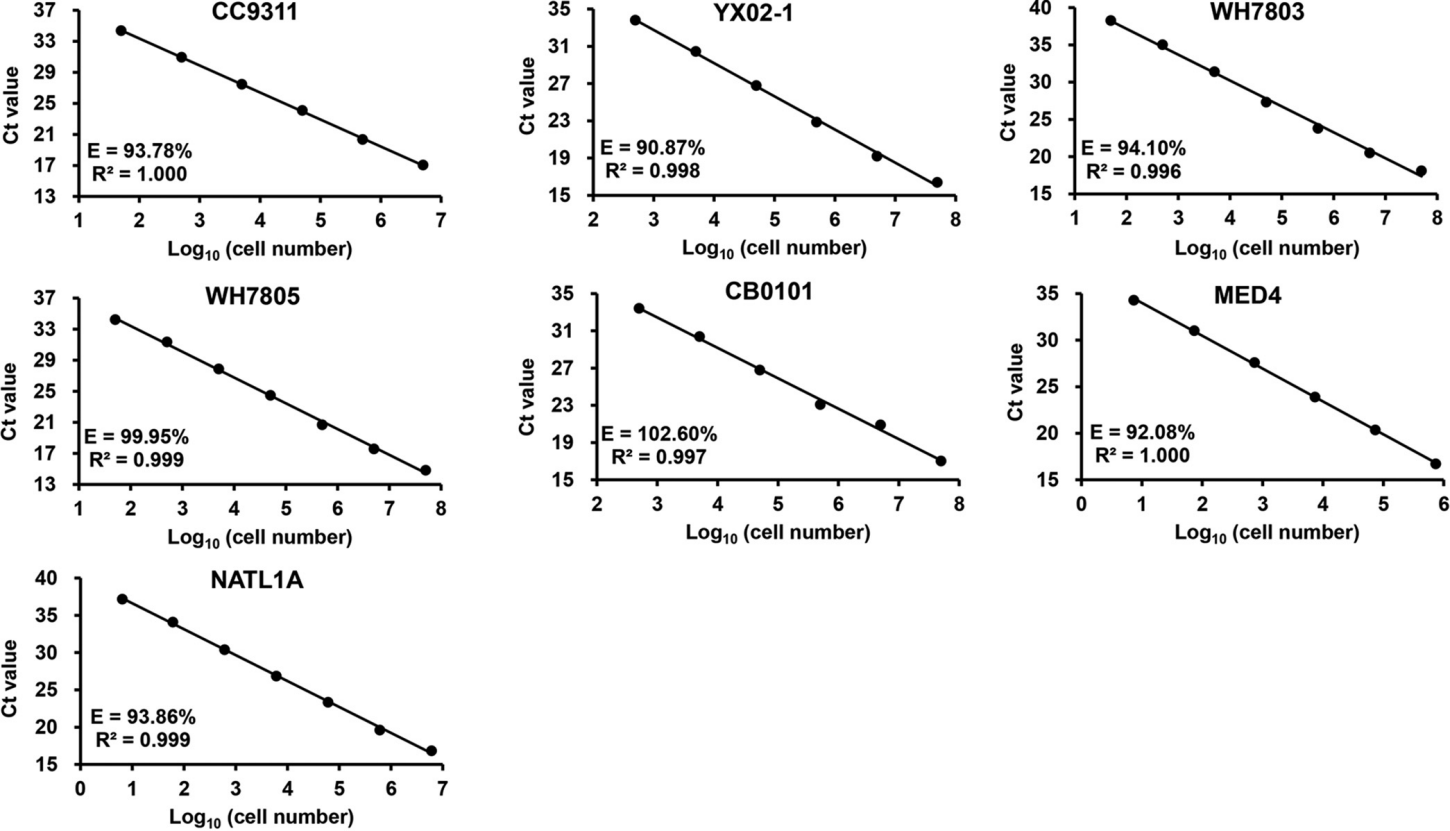
Amplification efficiency for different *Synechococcus* and *Prochlorococcus* strains.

To evaluate the amplification efficiency of this primer set on picocyanobacteria strains representing different ecotypes, we tested seven strains using their genomic DNA. The efficiency ranged from 90.87% to 102.6% (Fig. 4). No apparent difference was observed between *Prochlorococcus* and *Synechococcus* strains. Although amplicon size varied among different picocyanobacteria strains, the amplification efficiency did not appear to vary greatly.

**FIG 5:**
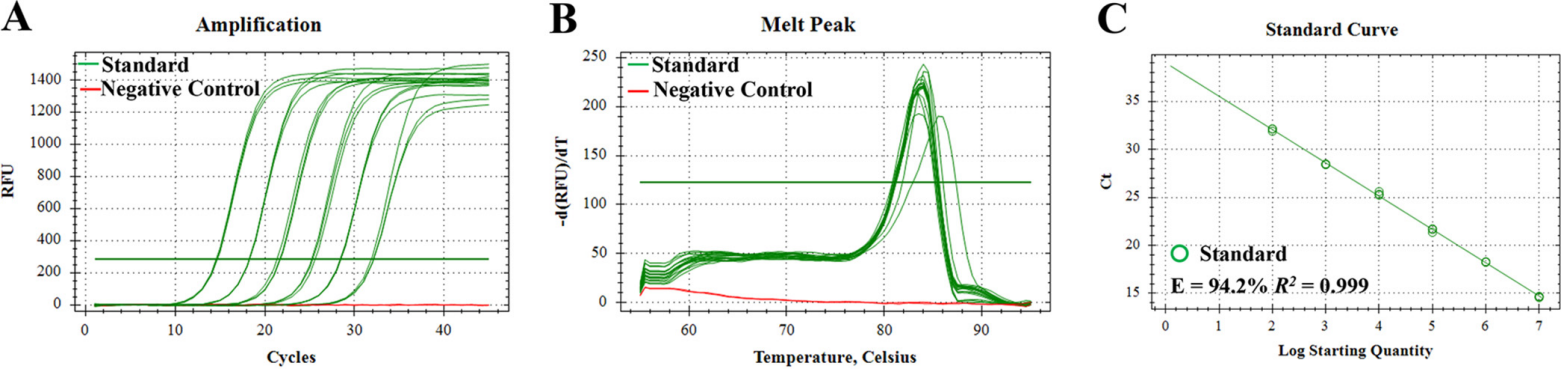
Amplification curve (A), melt peak (B), and standard curve (C). A mixed template prepared from nine *Synechococcus* and *Prochlorococcus* strains was used as the standard.

We mixed nine plasmid DNA with cloned ITS sequences as the template to establish a standard curve, which represented *Prochlorococcus* ecotypes HLI, HLII, LLI and LLIV and *Synechococcus* clades I, II, V, VI, and CB4 (Table 1). The amplification efficiency (92.9%) was determined using the mixed template (Fig. 5). No contamination, nonspecific amplification, or primer dimers were observed on the melt peak diagram (Fig. 5).

We tested the repeatability of the PCR assays using diluted environmental DNA. The variation coefficients were 0.29 to 0.77% within the same batch of reactions and were 0.69 to 0.85% among batches of reactions (Table S1). These results indicated acceptable repeatability and reproducibility.

10.1128/msphere.00499-22.5 TABLE S1 Verification of repeatability and reproducibility of qPCR assays on *C_T_* values. Download Table S1, DOCX file, 0.02 MB. Copyright © 2022 Zhang et al. 2022 Zhang et al. https://creativecommons.org/licenses/by/4.0/ This content is distributed under the terms of the Creative Commons Attribution 4.0 International license.

To test the detection limitation of this method, we measured serially diluted *Synechococcus* WH7803 cells with known cell density by qPCR. There was a strong linear relationship between threshold cycle (*C_T_*) values and the logarithm of cell numbers in the range from 14 to 1.4 × 10^5^ (Fig. S2). When the qPCR contained DNA equivalent to 1.4 cells and 1.4 × 10^6^ cells, the *C_T_* values showed great deviation from the linear regression. Therefore, the lower and upper detection limitations were 1.4 cells and 1.4 × 10^6^ cells, respectively. Samples outside the range should be concentrated or diluted.

10.1128/msphere.00499-22.2 FIG S2 Detection limitation of the qPCR assay. *Synechococcus* WH7803 cell stocks of cell numbers ranging from 1.4 to 1.4 × 10^6^ were used to test the detection limitation. Download FIG S2, TIF file, 0.1 MB. Copyright © 2022 Zhang et al. 2022 Zhang et al. https://creativecommons.org/licenses/by/4.0/ This content is distributed under the terms of the Creative Commons Attribution 4.0 International license.

### Comparison between qPCR and FCM for quantifying picocyanobacteria.

**FIG 6:**
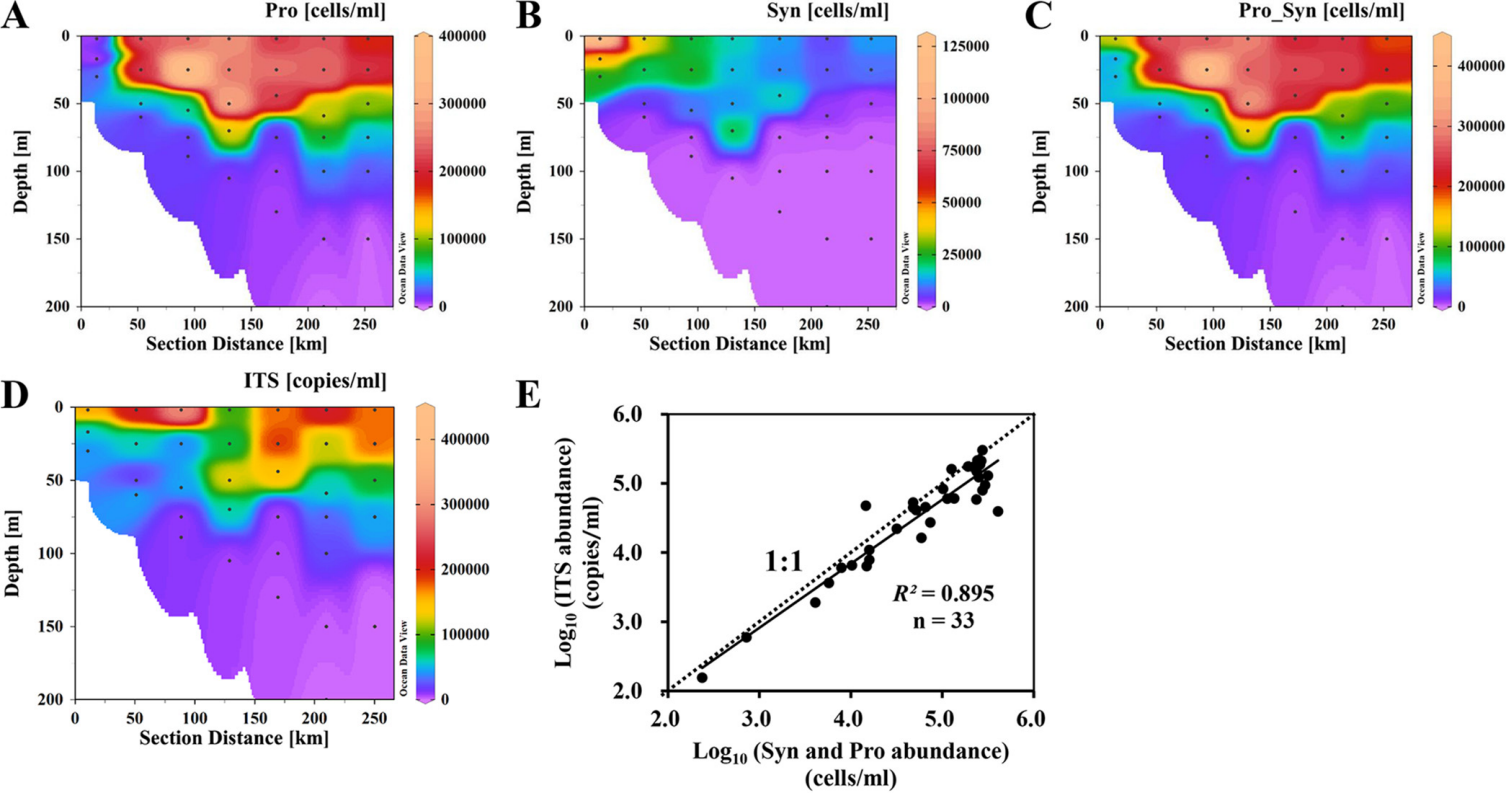
Picocyanobacterial abundance in the water column of a transect in the northern South China Sea measured by flow cytometry (A to C), qPCR (D), and a comparison between the two methods (E).

To further assess the qPCR method developed here, we compared the quantification of water samples by using qPCR and FCM. The analyzed samples were collected from a coastal-to-basin transect in the northern South China Sea (Fig. 1, stations 1 to 7). Along this transect, the distribution of *Prochlorococcus* and *Synechococcus* followed the typical pattern in which *Prochlorococcus* and *Synechococcus* were more abundant in offshore and nearshore waters, respectively (Fig. 6A and B). Measurement by qPCR and FCM generated a similar abundance pattern (Fig. 6C and D). Moreover, there was a strong linear relationship (*r* = 0.832) between the logarithms of abundances determined by qPCR and FCM (Fig. 6E). Nevertheless, the absolute abundances determined by qPCR were lower than those by FCM, despite marine *Synechococcus* possessing two identical copies of the ITS sequence. This could be due to losses during DNA extraction. Collectively, our result indicates this qPCR method could generate data comparable to FCM for water samples.

### Picocyanobacteria abundance on water column particles and in sediment.

**FIG 7:**
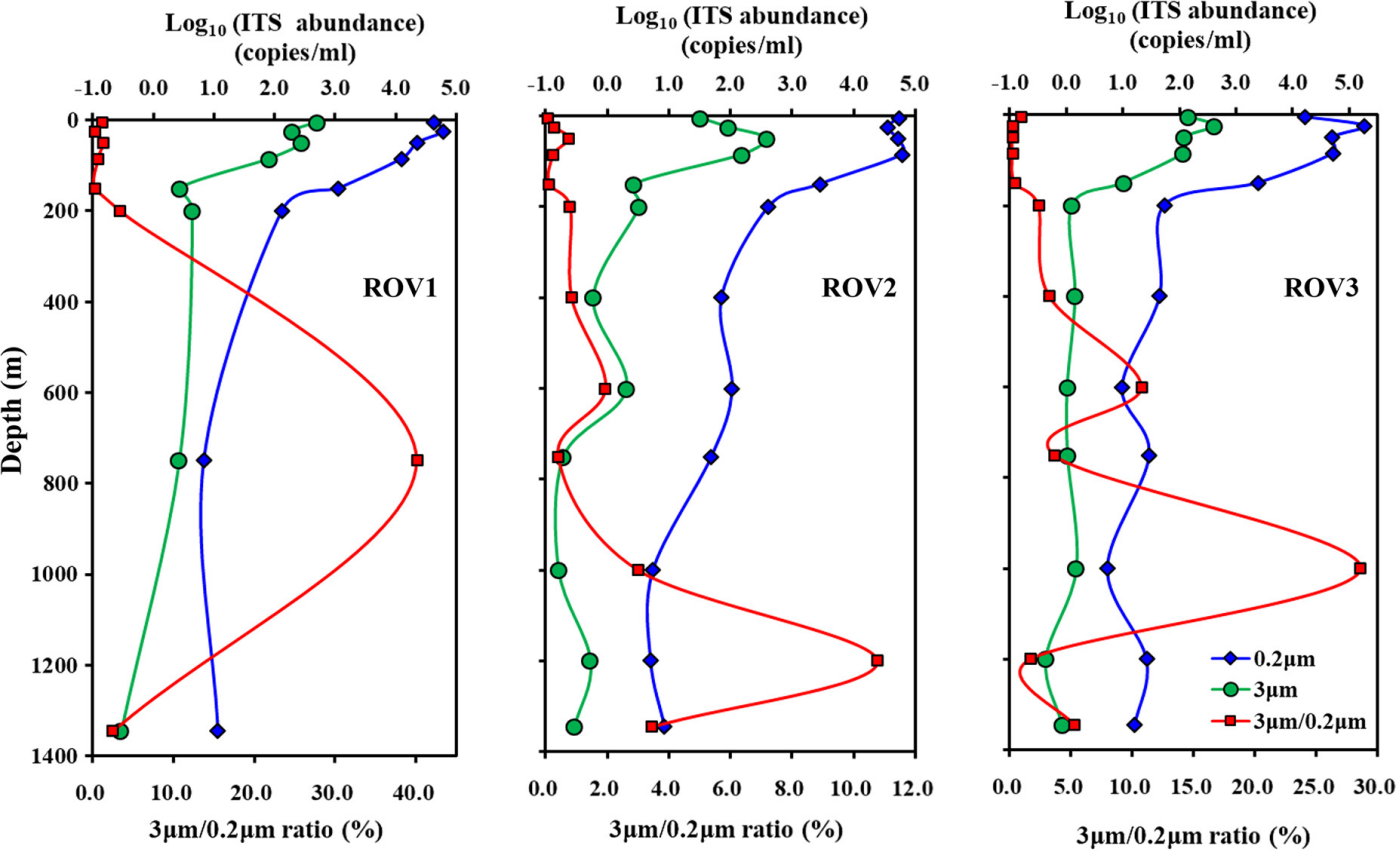
Picocyanobacterial ITS sequence abundance in different size fractions along water column profiles in the South China Sea.

We quantified the picocyanobacterial ITS sequence abundance in different size fractions of the water column at remotely operated vehicle (ROV) stations (Fig. 1). The ITS sequence abundance decreased from ~10^5^ copies/mL in the upper euphotic zone to ~10 copies/mL in the dark ocean in the size fraction of 0.2 to 3 μm (Fig. 7). In the size fraction of >3 μm, the ITS sequence abundances were 1 to 3 orders of magnitude lower than those in the size fraction of 0.2 to 3 μm. The ratio between the abundances in the >3-μm and 0.2- to 3-μm fractions increased along the depth profile, especially below 200 m (Fig. 7).

**FIG 8:**
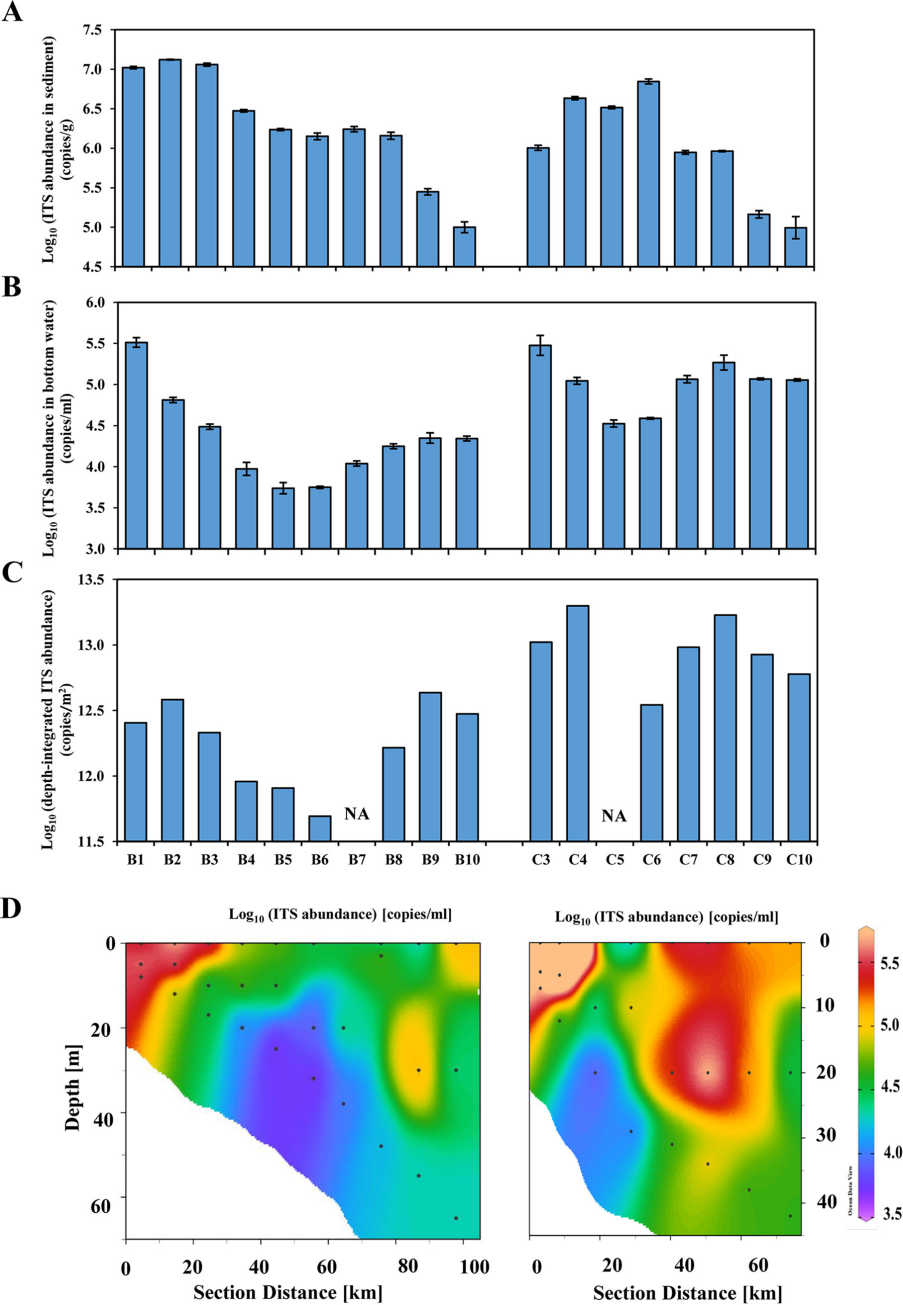
Picocyanobacterial ITS sequence abundance in the sediment (A) and water column (B to D) along two transects in the northern South China Sea as measured by qPCR. Error bars in panel A and panel B represent the standard deviation of values from three independent qPCRs.

The qPCR assay was also applied to investigate the picocyanobacteria abundance on water columns and in surface sediments along two nearshore-to-offshore transects in the northern South China Sea (see the sampling stations B1-B10 and C3-C10, shown in Fig. 1). The picocyanobacterial ITS sequence abundances ranged from 10^5^ to 10^7^ copies per gram of sediments in the two transects but appeared to have different trends. Along transect B, the ITS abundances gradually decreased, while in transect C, they increased first and then decreased (Fig. 8A). The ITS copies ranged from 5 × 10^3^ to 3 × 10^5^ per mL in the bottom water (Fig. 8B), and the depth-integrated abundance ranged from 5 × 10^11^ to 2 × 10^13^ copies per m^2^ (Fig. 8C). In contrast to the sediment samples, no similar patterns were observed in the bottom waters (Fig. 8B) or the whole water columns (Fig. 8C and D) along the nearshore-to-offshore transects.

## DISCUSSION

### Feasibility of the qPCR assay for detection of the marine *Synechococcus* and *Prochlorococcus* community.

*Synechococcus* and *Prochlorococcus* are the most abundant phototrophic organisms in the ocean (4). Epifluorescence microscopy and flow cytometry have been used to quantify their cell abundance for decades (2, 3, 15, 35). qPCR approaches were also developed to quantify numerous different *Synechococcus* and *Prochlorococcus* ecotypes at the population level (21, 26, 27). However, at the community level, a qPCR method to quantify marine picocyanobacteria as a whole was still lacking. The Syn/Pro clade of cyanobacteria includes both marine *Synechococcus* and *Prochlorococcus* and many nonmarine picocyanobacteria (6). This clade forms a robust, coherent phylogenetic group, which has been confirmed by phylogenomic analyses (36–38). To target this clade of cyanobacteria in marine environments, we designed a primer set based on the 16S-23S ITS sequence. The coverage and specificity of the primer set were evaluated by using the marine *Synechococcus* and *Prochlorococcus* strains and environmental samples, and a standard curve was established to quantify the ITS copies in field samples.

We used plasmids as standards to establish the standard curve between *C_T_* values and the decimal logarithms of ITS copy numbers instead of *C_T_* values and cell numbers as done in previous reports (21, 27). This is because we found that the extracellular DNA, cells, or cell detritus not detected by FCM in our cultures could greatly impact the calibration curve. Moreover, using plasmid standards would be more feasible for researchers who do not culture picocyanobacteria in their own laboratories. Furthermore, it is generally acknowledged by cyanobacterial molecular ecologists that ITS copies can vary in different cyanobacterial taxa. Therefore, here, we quantify the picocyanobacterial ITS copies rather than the exact cell numbers in natural samples. Due to the variation in DNA extraction efficiency, our measurement would slightly underestimate the cell abundance. Nevertheless, the quantification could well reflect the cell abundance in the field, based on comparison of the data generated by qPCR and FCM from the same set of water samples.

### Picocyanobacteria on water column particles and in sediments.

The qPCR assay was applied to quantify picocyanobacterial ITS sequence abundance in different size fractions in water columns. It was shown that, in the water column, picocyanobacteria standing stock are predominantly in the 0.2- to 3-μm size fraction, which likely represents free-living cells rather than those attached to large particles. This result suggests that the sinking of free-living picocyanobacteria cells may be an important form of carbon export to the deep ocean. The observation also supports the growing knowledge that small particles, like picocyanobacterial cells, contribute significantly to carbon export, especially in pico-/nanoplankton-dominated low-productivity regions (39–41). Nevertheless, the relative contribution of >3-μm particles to picocyanobacteria export likely increased along the depth profile, suggesting a more important role of larger particles in the deeper water.

We also measured marine picocyanobacterial ITS sequence abundance in sediments from the northern South China Sea by qPCR, and the abundance was in the range of 10^5^ to 10^7^ copies per gram. Although extracellular DNA was found to be a considerable fraction of nucleic acids in aquatic sediments, microbial 16S rRNA genes could not be amplified from extracted extracellular DNA (42). Therefore, we consider the ITS sequences detected in our sediment samples to be from cyanobacterial cells. The cell density of the picocyanobacteria standing stock in sediment appeared not to be related to the depth-integrated picocyanobacteria abundance in the water column (*R*^2^ = 0.046, *P > *0.1) (see Fig. S3A in the supplemental material) or the abundance in bottom water (*R*^2^ = 0.016, *P > *0.1) (Fig. S3B) on the date of sampling. In contrast, the correlations with the concentration of total carbon (*R*^2^ = 0.394, *P < *0.01) (Fig. S3C) and total nitrogen (*R*^2^ = 0.439, *P < *0.01) (Fig. S3D) in sediments were better. Such a relationship suggests a certain contribution of picocyanobacteria to the benthic carbon pool. The sediment picocyanobacteria abundances are likely greater than those in the water column by orders of magnitude for the same volume, that is, roughly, 1 g of sediment or 1 mL of water, indicating an accumulation of sinking picocyanobacteria in sediment. The standing stock of picocyanobacteria cells in sediments may be influenced by their sinking flux and resident time.

10.1128/msphere.00499-22.3 FIG S3 Relationship between picocyanobacterial ITS sequence abundance in sediment and depth-integrated ITS sequence abundance in the water column (A), ITS sequence abundance in bottom water (B), total carbon content (C), and total nitrogen content (D). Download FIG S3, TIF file, 0.2 MB. Copyright © 2022 Zhang et al. 2022 Zhang et al. https://creativecommons.org/licenses/by/4.0/ This content is distributed under the terms of the Creative Commons Attribution 4.0 International license.

Picocyanobacteria were thought to represent a negligible fraction of carbon export to the deep ocean due to their small cell size and, thus, slow sinking rate. However, they were often found in marine sediments (13), sinking particles (12, 43–46), and bathypelagic waters (23, 47). Moreover, the contribution of picoplankton to carbon export was found to be proportional to their total net primary production (14), suggesting an important role in the biological carbon pump of picocyanobacteria. Here, we provide a high-throughput molecular approach that quantifies the abundance of picocyanobacteria on water column particles and in sediments, which can be used to quantify the contribution of picocyanobacteria to the biological carbon pump in our future studies.

### Conclusion.

In this study, a new qPCR method for quantification of the marine picocyanobacteria was developed that is based on a newly designed primer set targeting the 16S-23S rRNA ITS sequence. The primer set covers a broad diversity of marine picocyanobacteria and avoids amplifying marine nitrogen-fixing cyanobacteria such as *Trichodesmium* and *Crocosphaera.* The method has good repeatability, reproducibility, and sensitivity. Using this approach, we found that free-living picocyanobacteria were much more abundant than the particle-associated cells in the dark ocean. We also found a high abundance of picocyanobacteria in surface sediments, suggesting an important role of picocyanobacteria in carbon export. Moreover, the newly designed primer set can also be used to investigate the diversity and community structures of marine picocyanobacteria via high-throughput amplicon sequencing. The qPCR and high-throughput amplicon sequencing method developed here may be applied to estimate the contribution of picocyanobacteria in marine biological carbon pumps.

## MATERIALS AND METHODS

### *Synechococcus* and *Prochlorococcus* cultures.

Five marine *Synechococcus* strains and two *Prochlorococcus* strains (Table 1) were cultured in the laboratory at 22°C under a light intensity of 10 μE m^−2^ s^−1^, with a light cycle of 14 h/10 h (light/dark). These strains represented different *Synechococcus* and *Prochlorococcus* ecotypes, which are abundant and widespread in marine environments. Lacking strains, we downloaded the ITS sequences of *Prochlorococcus* strains MIT9312 and MIT9313 from the NCBI website and synthesized them at TsingKe Biological Technology (Beijing). Trichodesmium erythraeum IMS101 and Crocosphaera watsonii WH8501 were shared by Dalin Shi at Xiamen University, China. Freshwater cyanobacteria strains FACHB-1061, FACHB-410, FACHB-2210, FACHB-800, and FACHB-920 (Table 1) were bought from Freshwater Algae Culture Collection at the Institute of Hydrobiology (FACHB).

### Sampling.

Seawater samples were collected from Sanya Bay stations W4 (18°12′N, 109°25′E) and W6 (18°15′N, 109°25′E) (October 2014) in the surface layer, Indian Ocean station I205 (0°0′N, 87°59′E, onboard RV *Shiyan I*, March 2015) in different water depths, four transects in the northern South China Sea (onboard RV *Shiyan III*, September 2018, and *YueZhanYuKe 10*, July 2019), and the ROV stations in the South China Sea (onboard RV *HaiYangDizhi IV*, September 2020) (Fig. 1). Sediment samples were collected using a Peterson's grab sampler at each site from two transects of the northern South China Sea (onboard RV *YueZhanYuKe 10*, July 2019) (Fig. 1). Seawater (1 to 1.5 L) was filtered through 0.22-μm-pore-size polycarbonate filter membranes (Millipore) to harvest microbial cells. For samples collected at the ROV stations, seawater was prefiltered through 3-μm-pore-size polycarbonate filter membranes (Millipore). The membrane and the sediment samples were stored at −80°C until DNA extraction. To enumerate the cell abundance of *Synechococcus* and *Prochlorococcus* by flow cytometry, 1.96 mL seawater was fixed with 40 μL glutaraldehyde (50% [vol/vol]) for 15 min in the dark. The fixed samples were immediately frozen in liquid nitrogen and stored at −80°C. Total carbon and total nitrogen content in sediments were measured following previously described methods (48).

### DNA extraction.

Environmental DNA from the filter membranes was extracted using the PowerSoil DNA isolation kit (Mo Bio Laboratories) for stations W4, W6, and I205 and the DNeasy PowerWater kit (Qiagen) for all other membrane samples. The FastDNA Spin kit for soil (MP) was used to extract DNA from sediment samples (~0.5 g). The PureLink genomic DNA minikit (Invitrogen, Thermo Fisher Scientific) was used to extract genomic DNA from cultured *Synechococcus* and *Prochlorococcus* cells. All extraction procedures followed the manufacturer’s protocols. The environmental DNA was eluted in 100 μL elution buffer.

### Primer design.

A primer set was designed based on the 16S-23S rRNA ITS sequences of marine *Synechococcus* and *Prochlorococcus* strains. The sequences were aligned with Clustal X2 (49), and the alignment was examined by eyes to find conserved motifs. The forward primer (5′-GCTTTGCAAGCAGGATGTCAG-3′) and reverse primer (5′-CTATGCAGTTGTCAAGGTTC-3′) target the tRNA-Ala and the antitermination Box A motif, respectively.

### Assessing qPCR condition and amplification efficiency.

The qPCR cycling program consisted of an initial step at 95°C for 30 s, 45 cycles of amplification, including three steps of 95°C for 5 s, annealing temperature, for 30 s, and 72°C for 45 s, and a melting curve step with a temperature gradient from 65°C to 95°C. The annealing temperature was optimized with a temperature gradient from 53°C to 63°C using environmental DNA (extracted from a sample in this study) as the template. The 25-μL reaction mixture contained 12.5 μL premix, 1 μL of each primer (working solution concentration, 10 μM), 1 μL template DNA, and 9.5 μL H_2_O. The qPCRs at the annealing temperature of 61.3°C had the lowest *C_T_* values (see Fig. S4 in the supplemental material), suggesting that the qPCR assay was more sensitive at this annealing temperature. Therefore, we set the annealing temperature at 61.3°C. Amplification efficiency was evaluated by testing the genomic DNA of seven *Synechococcus* and *Prochlorococcus* strains (Table 1). Genomic DNA was 10-fold serially diluted, and qPCR assays were performed in triplicate for each strain. Amplification efficiency was calculated using the CFX Manager software version 3.1 (Bio-Rad).

10.1128/msphere.00499-22.4 FIG S4 Comparison of *C_T_* values under a gradient of annealing temperature from 53°C to 63°C using mixed environmental DNA from the Indian Ocean station I205. Download FIG S4, TIF file, 0.1 MB. Copyright © 2022 Zhang et al. 2022 Zhang et al. https://creativecommons.org/licenses/by/4.0/ This content is distributed under the terms of the Creative Commons Attribution 4.0 International license.

### Investigating picocyanobacterial diversity with Sanger and high-throughput sequencing.

Two environmental DNA samples were mixed (stations W4 and W6 in the Sanya Bay, 5 m and 25 m at Indian Ocean station I205, 50 m and 75 m at I205, and 100 m and 150 m at I205) with equivalent volume, and the four mixed DNA composites were used to amplify the ITS sequence with the optimized qPCR conditions as described above. qPCR products were gel purified using the E.Z.N.A. gel extraction kit (Omega Bio-Tek) and cloned using the pMD18-T vector kit (TaKaRa). A total of 147 clones were sequenced from the 4 clone libraries. The obtained environmental ITS sequences were aligned with reference sequences using Clustal X2, and a phylogenetic tree was constructed using MEGA7. The alignment was manually examined to identify nonspecific amplification. The maximum-likelihood (ML) method based on the Jukes-Cantor model was used to estimate the phylogeny. iTOL (50) was used to visualize the tree.

The primers were barcoded for amplicon high-throughput sequencing. The 20-μL reaction mixture contained 0.1 μL Pro Taq high-sensitivity (HS) DNA polymerase (Accurate Biotechnology [Hunan] Co., Ltd.), 2 μL 10× Pro Taq PCR buffer (Mg^2+^ plus), 0.4 μL deoxynucleoside triphosphate (dNTP) mix (10 mM each), 0.4 μL primer (10 μM) for each, and 10 ng template DNA. The amplification was conducted according to the following cycle parameters: denaturation for 3 min at 95°C, 30 cycles of 95°C for 30 s, 61.3°C for 30 s, and 72°C for 45 s, and a final 10-min elongation at 72°C. Samples were from stations 8, 10, 12, and 14 in the South China Sea. The PCR product was extracted from 2% agarose gel and purified using the AxyPrep DNA gel extraction kit (Axygen Biosciences, Union City, CA, USA) according to the manufacturer’s instructions and quantified using Quantus fluorometer (Promega, USA). Pooled products were sequenced using a MiSeq 300-bp paired-end platform by Majorbio Bio-Pharm Technology Co. Ltd. (Shanghai, China).

We collected 1,699 ITS sequences (within the Syn/Pro clade) from picocyanobacteria strains and environmental samples on the NCBI website (34, 51–63). Because a few cyanobacteria not belonging to the Syn/Pro clade could be amplified using the primer set we designed (Table 1), we also collected 669 ITS sequences from cyanobacteria not belonging to the Syn/Pro clade. The ITS sequences were truncated to match the amplicon region. A phylogenetic tree was built based on the 1699 ITS sequences within the Syn/Pro clade, using QIIME2 with the command “phylogeny align-to-tree-mafft-fasttree”. Ecotypes were assigned to each of the sequences (within the Syn/Pro clade) based on their position on the tree and previous literature on these sequences. Reference sequences not belonging to the Syn/Pro clade were assigned to the non-Syn/Pro clade. This database was used in the pipeline for analyzing high-throughput amplicon sequencing data and was accessible at FigShare (https://figshare.com/articles/dataset/cyanobacteria_ITS_reference_database_v1_8/20963053).

Quality filtering for raw reads was conducted using fastp (64) as follows: (i) reads were truncated at any site receiving an average quality score of <20 over a 50-bp sliding window, (ii) truncated reads shorter than 50 bp were discarded, and (iii) reads containing ambiguous characters were removed. Paired-end reads were merged using FLASH (65) with the parameters minimum overlapping region of 10 bp and maximum mismatch ratio of 0.2. Reads that cannot be assembled were removed. QIIME2 was used to process the following analyses. First, assembled reads were denoised, and amplicon sequence variants (ASVs) were clustered with DADA2. Second, the sequences of ASVs were filtered by aligning to the reference sequences in the above-described database with the parameters as: identity >70% and coverage >50% of queries. Third, the retained reads after filtration were assigned to picocyanobacteria ecotypes at 70% confidence by comparing them to the reference database. Last, reads which were assigned to non-Syn/Pro clade were removed. The codes running in QIIME2 were provided at FigShare (https://figshare.com/articles/dataset/QIIME2_codes_for_cyanobacterial_ITS_sequence_analysis_v2/20963515).

### Standard curve.

ITS sequences were amplified from the nine *Synechococcus* and *Prochlorococcus* strains (Table 1) using the qPCR condition described above. qPCR products were gel purified and cloned into the pMD18-T vector, and five clones for each strain were screened by PCR with the primers M13F (5′-TGTAAAACGACGGCCAGT-3′) and M13R (5′-CAGGAAACAGCTATGACC-3′). One positive clone for each strain was grown in 5 mL LB medium, and plasmid DNA was extracted using the E.Z.N.A. plasmid minikit I (Omega Bio-Tek). DNA fragments were amplified using the primers M13-F and M13-R with plasmid DNA as the template. PCR products were gel purified, and the DNA concentration was determined using Qubit 3 with the reagent from Invitrogen. The purified DNA was 10-fold serially diluted and served as qPCR standard. Each of the diluted standards with molecular concentrations from 10^1^ to 10^7^ copies/μL was measured in triplicate. The standard curve was generated by using the CFX Manager software.

### Assessing repeatability and reproducibility.

To assess repeatability of the method, 10 replicates of qPCRs were performed at 3 different concentrations of DNA template. Then, another two batches of qPCRs were performed with the same DNA template at different times to test the reproducibility of the method.

### Assessing detection limitation.

*Synechococcus* strain WH7803 was 10-fold serially diluted to prepare cell stocks with known cell density. The cell stocks were mixed with Chelex 100 resin (Bio-Rad; final concentration 5% [wt/vol]) and heated at 100°C for 10 min to lyse the cells. The mixture was then centrifuged at 10,000 × *g* for 10 min, and the supernatant was used as the template for the qPCR assay. The DNA templates were equivalent to 1.4 × 10^6^, 1.4 × 10^5^, 1.4 × 10^4^, 1.4 × 10^3^, 1.4 × 10^2^, 1.4 × 10^1^, and 1.4 cells. There are two identical copies of the ITS sequence in the WH7803 genome.

### *Synechococcus* and *Prochlorococcus* cell enumeration by flow cytometry.

Cell concentrations were measured by a CytoFlex S flow cytometer (Beckman Coulter). Red fluorescence and forward angle light scattering properties were used to identify the *Prochlorococcus* cells. *Synechococcus* cells were identified using side scatter and orange fluorescence for phycoerythrin-rich cells and red fluorescence for phycocyanin-rich cells. Cell gating and counting were performed using the CytExpert software version 2.4.

### qPCR assay for environmental samples.

The ITS sequence abundance of environmental DNA was measured using the qPCR condition described above. qPCRs were performed in triplicate for each DNA sample. The number of molecules in the reaction was calculated according to the standard curve. The molecular concentration in the field samples was calculated by considering the volume of water samples or weight for sediments and the elution volume of DNA.

### Statistical analyses.

Nonmetric multidimensional scaling analysis (NMDS) was carried out to demonstrate the β-diversity of picocyanobacteria among depths in different stations with Bray-Curtis similarity matrices using the vegan package in R statistical software (version 4.1.0). Analysis of similarities (ANOSIM) was used to evaluate the significance of grouping among samples with 999 iterations. Sampling depth was considered a factor and tested in ANOSIM. The data were logarithmically transformed before analyzing the variation of ITS sequence abundance with depth and station. The depth-integrated abundance of ITS sequences was also analyzed, which integrated samples from the entire water column, rather than from a single or fixed depth, and can represent the sample more accurately. As none of the data (qPCR and FCM) followed the normal distribution, Spearman’s correlation analysis was conducted to assess the relationship between them.

### Data availability.

DNA sequences for the clone library were deposited into GenBank under accession numbers MW374331 to MW374458, and those for high-throughput sequencing were deposited into SRA under accession no. PRJNA750845. The cyanobacterial 16S-23S ITS reference database is available at FigShare at https://figshare.com/articles/dataset/cyanobacteria_ITS_reference_database_v1_8/20963053, and QIIME2 codes can be accessed at https://figshare.com/articles/dataset/QIIME2_codes_for_cyanobacterial_ITS_sequence_analysis_v2/20963515.
