# Supplementary figures and images for: Quantification of Marine Picocyanobacteria on Water Column Particles and in Sediments Using Real-Time PCR Reveals Their Role in Carbon Export
Source: mSphere. 2022 Dec 6;7(6):e00499-22. doi: 10.1128/msphere.00499-22 (PMC9769826; doi:10.1128/msphere.00499-22)

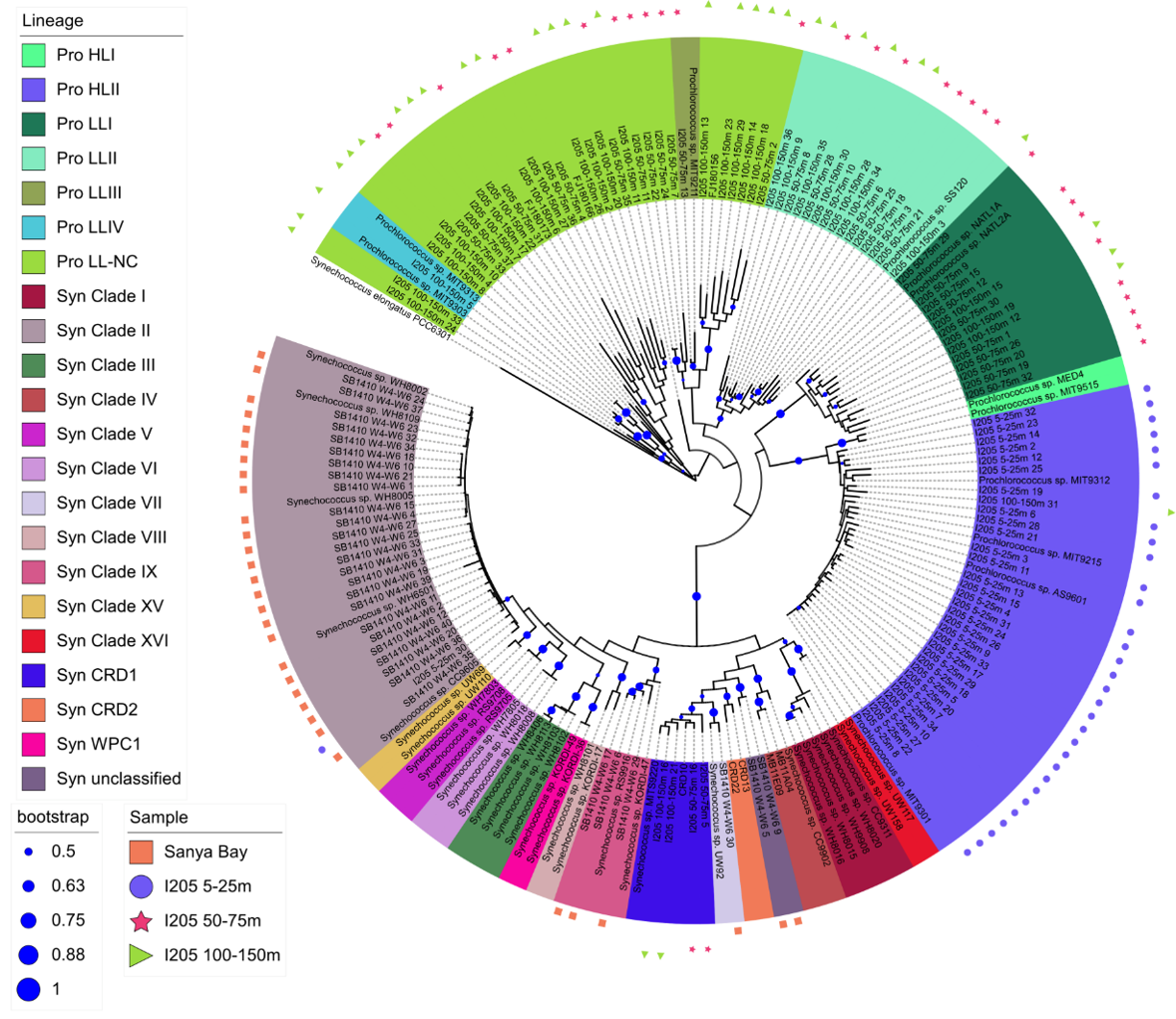

Supplement: FIG S1 [file msphere.00499-22-s0002.tif]

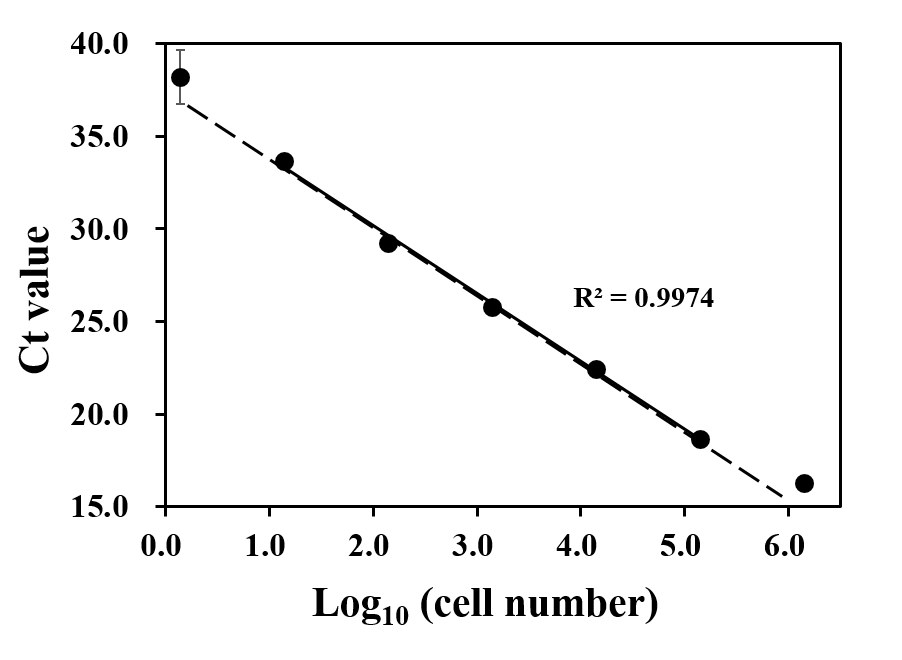

Supplement: FIG S2 [file msphere.00499-22-s0003.tif]

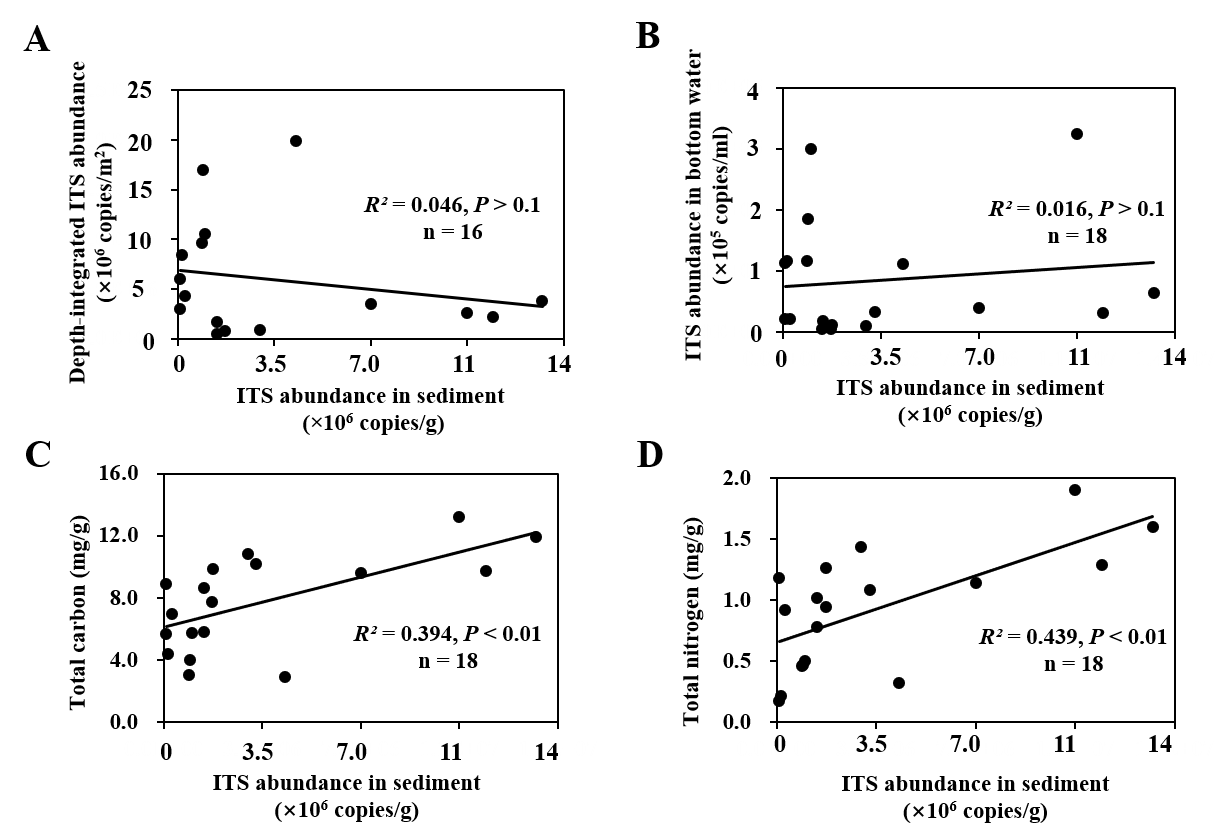

Supplement: FIG S3 [file msphere.00499-22-s0004.tif]

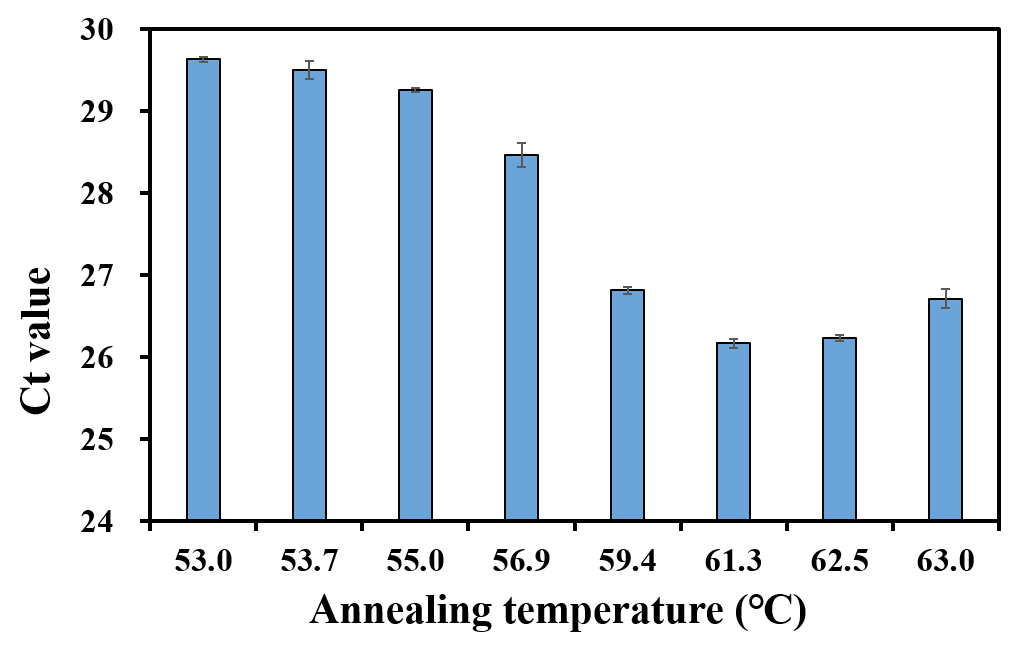

Supplement: FIG S4 [file msphere.00499-22-s0005.tif]
