# Supplementary material for: Quantification of Marine Picocyanobacteria on Water Column Particles and in Sediments Using Real-Time PCR Reveals Their Role in Carbon Export
Source: mSphere. 2022 Dec 6;7(6):e00499-22. doi: 10.1128/msphere.00499-22 (PMC9769826; doi:10.1128/msphere.00499-22)
Supplement: TABLE S1 [file msphere.00499-22-s0001.docx]

**Table S1** Verification of repeatability and reproducibility of qPCR assays on Ct values.

| **Batch of qPCR reactions** | **High concentration** | |  | **Medium concentration** | |  | **Low concentration** | |
| --- | --- | --- | --- | --- | --- | --- | --- | --- |
|  | **Ct ± SD** | **Variable coefficient** |  | **Ct ± SD** | **Variable coefficient** |  | **Ct ± SD** | **Variable coefficient** |
| Within batch1 | 18.21±0.05 | 0.29% |  | 26.55±0.17 | 0.63% |  | 32.21±0.25 | 0.77% |
| Within batch2 | 18.10±0.07 | 0.36% |  | 26.33±0.17 | 0.63% |  | 31.77±0.20 | 0.62% |
| Within batch3 | 18.35±0.10 | 0.53% |  | 26.36±0.17 | 0.64% |  | 32.12±0.14 | 0.44% |
| Among three batches | 18.22±0.13 | 0.69% |  | 26.42±0.19 | 0.72% |  | 32.04±0.27 | 0.85% |
